# Labeling of Baropodometric Analysis Data Using Computer Vision Techniques in Classification of Foot Deformities

**DOI:** 10.3390/medicina59050840

**Published:** 2023-04-26

**Authors:** Siniša S. Babović, Mia Vujović, Nebojša P. Stilinović, Ostoja Jeftić, Aleksa D. Novaković

**Affiliations:** 1 Department of Anatomy, Faculty of Medicine, University of Novi Sad, 21000 Novi Sad, Serbia; 2 Chair of Telecommunications and Signal Processing, Faculty of Technical Sciences, University of Novi Sad, 21000 Novi Sad, Serbia; 3 Department of Pharmacology, Toxicology and Clinical Pharmacology, Faculty of Medicine, University of Novi Sad, 21000 Novi Sad, Serbia

**Keywords:** arch index, segmentation, machine learning, baropodometry

## Abstract

*Background and Objectives*: Foot deformities are the basis of numerous disorders of the locomotor system. An optimized method of classification of foot deformities would enable an objective identification of the type of deformity since the current assessment methods do not show an optimal level of objectivity and reliability. The acquired results would enable an individual approach to the treatment of patients with foot deformities. Thus, the goal of this research study was the development of a new, objective model for recognizing and classifying foot deformities with the application of machine learning, by labeling baropodometric analysis data using computer vision methods. *Materials and Methods*: In this work, data from 91 students of the Faculty of Medicine and the Faculty of Sports and Physical Education, University of Novi Sad were used. Measurements were determined by using a baropodometric platform, and the labelling process was carried out in the Python programming language, using functions from the OpenCV library. Segmentation techniques, geometric transformations, contour detection and morphological image processing were performed on the images, in order to calculate the arch index, a parameter that gives information about the type of the foot deformity. *Discussion*: The foot over which the entire labeling method was applied had an arch index value of 0.27, which indicates the accuracy of the method and is in accordance with the literature. On the other hand, the method presented in our study needs further improvement and optimization, since the results of the segmentation techniques can vary when the images are not consistent. *Conclusions*: The labeling method presented in this work provides the basis for further optimization and development of a foot deformity classification system.

## 1. Introduction

In spite of the high incidence of foot deformities in population [1], they are not treated with an appropriate amount of attention. Foot deformities impair one of its basic functions—maintaining the physiological relation of static load and its transfer to the ground. The foot is a key element in human biomechanics, because it has the following functions [2]: it maintains an appropriate weight distribution and transfer to the ground, it supports the body, and it allows for the maintenance of posture without extensive muscle use. The foot adapts to the irregularities of surfaces while standing and walking. It behaves like a spring and therefore can be separated from surfaces while walking.

The statics of the foot include the influence of all forces that affect the foot while standing. The trabecular system of bones follows the direction of these forces. One of these forces is the weight of one’s body, by which it applies pressure on a surface. About 45% of the force is transferred to the surface via the front part of the foot and the rest via the rear part of the foot [2,3,4]. According to Newton’s third law, there are forces by which the surface reactively affects the foot while it is on the ground. These are called ground reaction forces and are crucial in the development of deformities of the locomotor apparatus [5,6]. From the aspect of dynamics, the foot is a second-class lever, which means that the resistive force to the weight of one’s body is located between the fulcrum (the heads of the metatarsal bones) and the effort (*tendo calcaneus*—the Achilles tendon) [7].

During gait, the foot rotates in relation to the line of progression, which is a sagittal plane located along the direction of one’s gait. The rotation of the foot occurs in the horizontal plane. The angle which is formed by the line of progression and the long axis of the foot (the line which connects the calcaneal and second metatarsal bone) is known as the foot progression angle (FPA). Based on the FPA during gait, one can determine whether the foot rotates inwards, which is called in-toeing, or outwards, which is called out-toeing, and if this rotation is within the reference range [8,9,10].

In the case of certain foot deformities (flat feet, metatarsalgia, hollow feet, painful heel, etc.), a non-physiological load distribution occurs. Early recognition and treatment of such pathological conditions is important, because otherwise damage to other parts of the locomotor apparatus occurs. One of the most important deformities is flat feet (*pes planus*). It is a static collapse of the foot as a consequence of the inability to passively maintain the mutual morpho-functional relations of the foot bones. Complications that arise as a result of flat feet are as follows: inflammation of the Achilles tendon and the posterior tibial muscle tendon; bunions; hammer toes; plantar fascitis; arthritis of the ankle and foot; and pain in the foot, calf, knee joint, hip and the lumbosacral part of the spine. Considering that flat feet significantly reduce the degree of the absorption of shock waves when walking, this condition can lead to pericarditis as well [11,12]. Another example of deformities is hollow feet (*pes cavus*). It is characterized by high longitudinal arches, caused by damage to muscles or neuromuscular synapses. It is described as a fixed *pes equinus* of the forefoot towards the hindfoot. Complications of this deformity are as follows: ankle instability, knee pain, iliotibial band friction syndrome and tripping [13].

In the diagnostics of foot deformities, the most important test is the adequate determination of the height of the internal longitudinal arch of the foot (it is partially or completely absent in flat feet, while it is abnormally high in hollow feet) [14]. Some of the methods still in use are as follows: visual observations, radiographs, making footprints with ink and others. The disadvantage of all the mentioned techniques is that they are either extremely expensive, or insufficiently objective and precise. Therefore, a new measure was introduced—the arch index (AI), which is based on the area of the middle part of the foot and is simply calculated [15,16,17]. Numerous studies in which the AI was determined from ink footprints have shown a high correlation coefficient of −0.7 between the height of the inner longitudinal arch of the foot and the AI, making it the most reliable parameter for diagnosing foot deformities so far. Although this method is inexpensive and non-invasive, it is uncomfortable for the patient, time-consuming, non-automated and highly dependent on the examiner [18]. On the other hand, machine learning, as an integral part of artificial intelligence, can extract meaningful information from images, videos, and other visual inputs, allowing it to correctly identify various medical conditions [19]. Thus, we proposed that due to the development of baropodometric platforms and machine learning in the field of computer vision, it is possible to automate the AI calculation method, making it quicker, more precise, objective, and more pleasant for the patient, while keeping it inexpensive and non-invasive.

## 2. Technique

All participants were given detailed instructions regarding the technique used and all of them signed written informed consent for participation in measurements, which were performed at the Faculty of Medicine. Ethics committee of the Faculty of Medicine gave written approval for this research (approval No. 01-39/3/1/2, date: 7 February 2019).

For examining patients’ feet, we used the baropodometric platform *freeMed Maxi* with included software *freeStep v.1.4.01* (Sensor Medica, Rome, Italy). The platform was equipped with pressure sensors that can determine biometric parameters regarding the statics and dynamics of the foot. Measured values were transferred to a PC with installed *freeStep* program via USB connection. Numerical values of the parameters were shown in the program, as well as an isochromatic map of the sole, determined by its load. Alongside with the measured values, software displayed physiological values with a reference range recommended by the manufacturer. Still, it should be noted that the goal of this technical method was not a discussion of footprint indices or a comparison between them.

The participants’ height, weight and shoe size were determined. These values were inserted into the program. The participants then stepped on the baropodometric platform with both of their feet and stood still. The participants remained in this position for several seconds, until the machine was finished reading the data. The participants then walked in one direction, during which they stepped on the platform only with their right foot. Afterwards, they walked in the opposite direction, again stepping on the platform only with their right foot. Then the process was repeated, but instead the participants stepped on the platform only with their left foot. This means that for each participant, one static and two dynamic prints for each foot were recorded. However, for further analyses, static prints of the feet were used.

The output data of the static analysis of each participant were in .pdf format, from which the images (in .jpg format) were cut, which were then used to label the data in this paper. The labeling was carried out in the Python programming language, in which functions from the OpenCV library were mainly used, as well as the Imutils, Math and NumPy libraries. 

**Figure 1:**
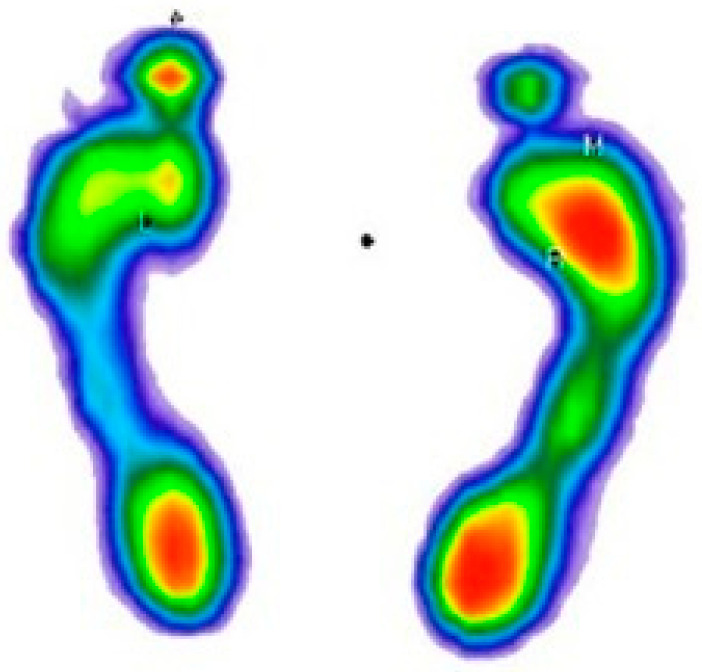
Display of loaded image. The color map of the pressures: red to dark green—from the area of the highest to the lowest level of pressure; blue—foot perimeter.

After manually cutting the images from the .pdf document, the next step was to upload the images (Figure 1). For this step of the process, only one person was responsible in order to avoid inter-rater differences. 

**Figure 2:**
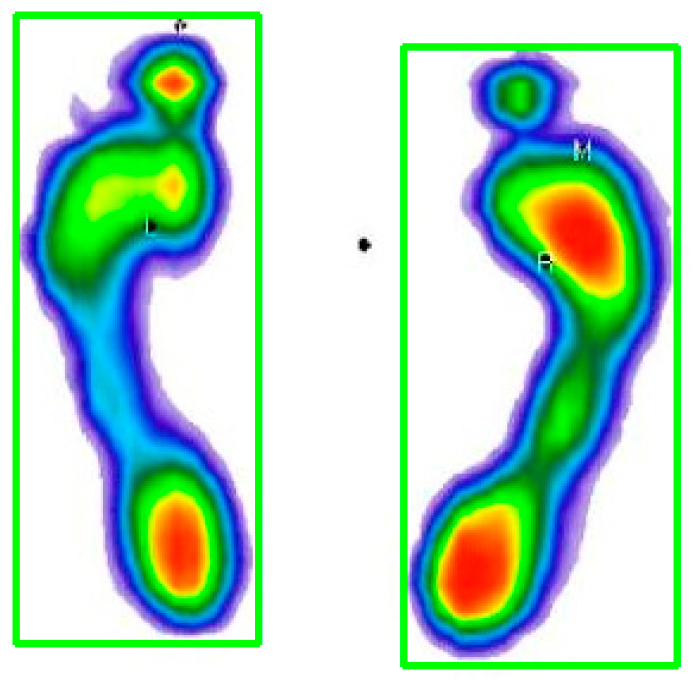
Contour detection and rectangle generation. The color map of the pressures: red to dark green—from the area of the highest to the lowest level of pressure; blue—foot perimeter.

The first technique that was applied was the detection of foot contours, whereby the two largest contours in the image were detected, that is, the contours of both feet separately. Contour detection was performed based on color segmentation, after which segment contours were detected. Based on the contours, enclosing rectangles were generated so that the feet could be separated (singled out) (Figure 2).

**Figure 3:**
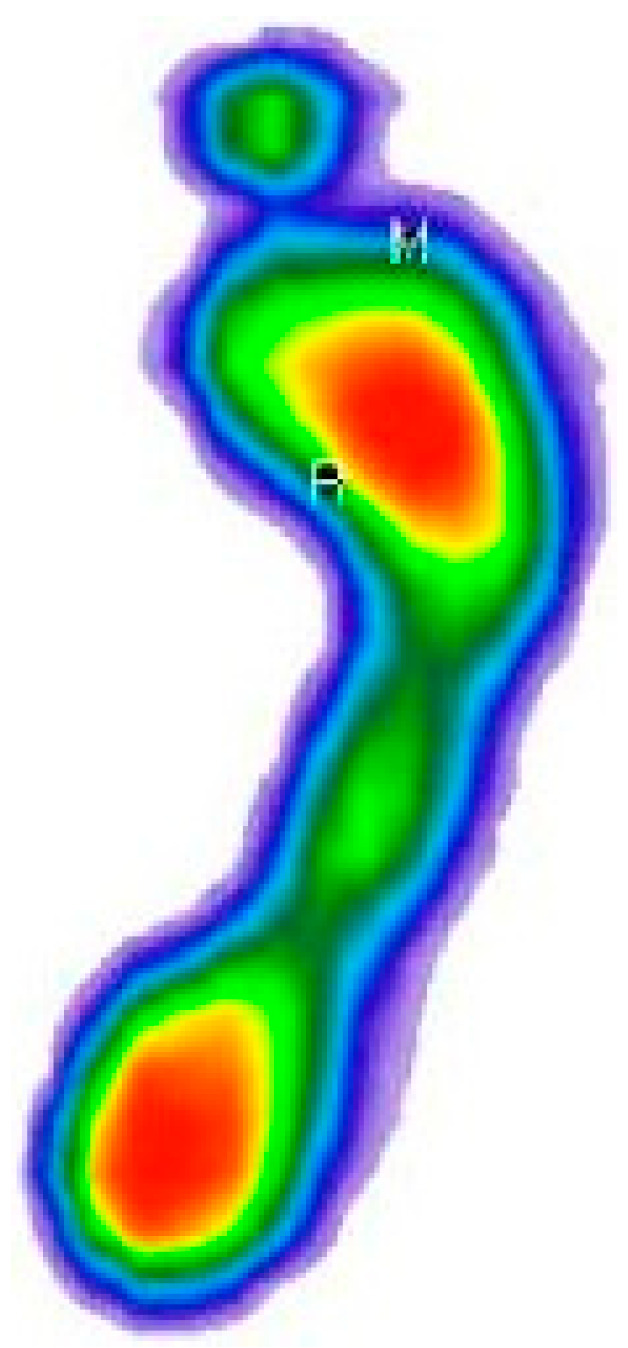
Right footprint segmented by a rectangle. The color map of the pressures: red to dark green—from the area of the highest to the lowest level of pressure; blue—foot perimeter.

Then the footprints were segmented by a rectangle, which can be seen in Figure 3.

**Figure 4:**
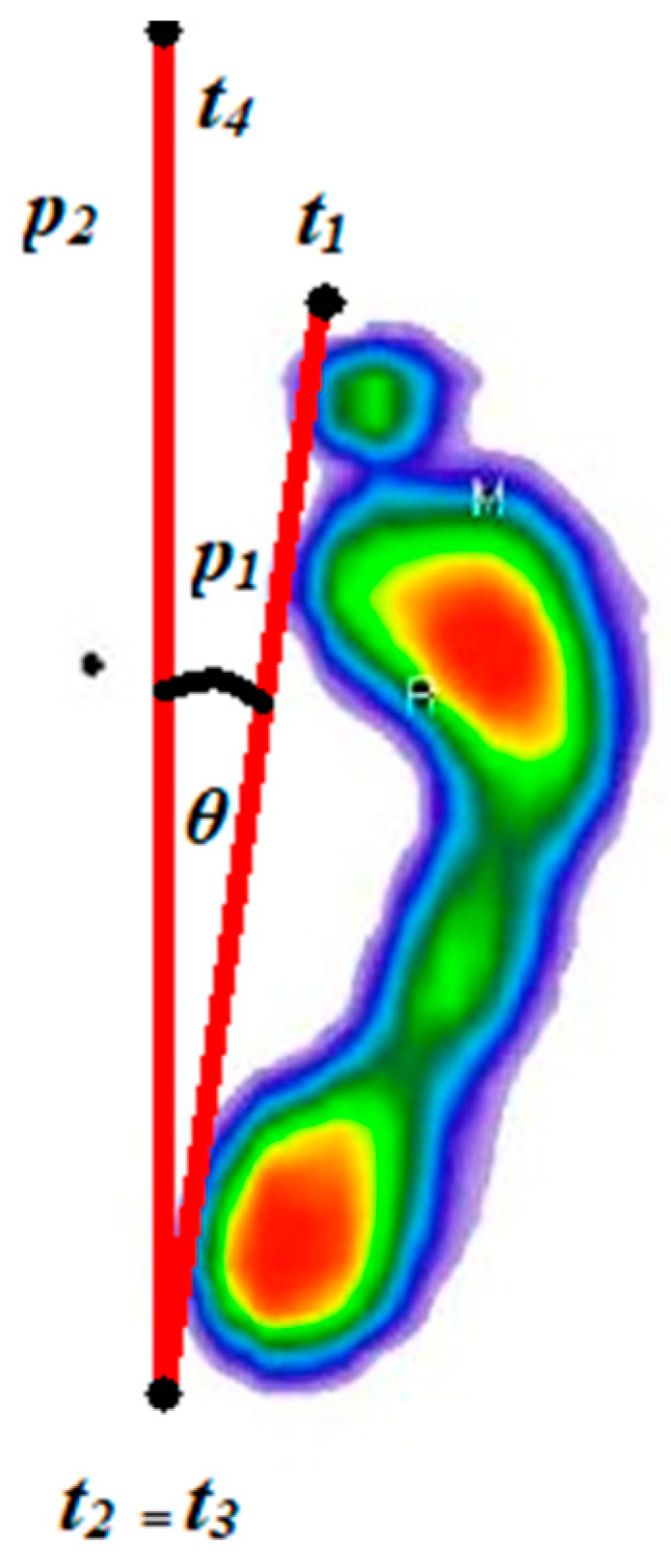
Display of foot progression angle (*θ*) and geometric parameters. The color map of the pressures: red to dark green—from the area of the highest to the lowest level of pressure; blue—foot perimeter. Legend: t1 and t 2 are the points of the p1 line (longitudinal axis of foot); t3 and t4 are the points of line p2 line (gait progression axis).

After that, it was necessary to rotate the foot by the foot progression angle (*θ*), and the lines that make up that angle had to be determined based on the coordinates of the points that define those lines. The first line (p1) was determined by two more medial points in relation to the longitudinal axis of the foot, that is, two points representing the minimum (t1) and maximum values of the rectangular contour of the foot along the y-axis (t2). The second line (p2) was defined based on the point (t3), which is at the same position as the point (t2) and the zero value of the y-axis (t4). The resulting angle can be seen in Figure 4.

The coordinates of point t1 were determined as follows: $t1\left({x1}_{avg}{,min}_{yd}\right)$, where ${min}_{yd}$ is minimum foot contour value along the y-axis, and ${x1}_{avg}$ is calculated in the following way:

(1) $${x1}_{avg}=\frac{{min}_{xd}-{max}_{yd}}{2}$$

where ${min}_{xd}$ is minimum foot contour value along the x-axis, and ${max}_{yd}$ is maximum foot contour value along the y-axis.

The coordinates of points t2 = t3 were determined as follows: $t2\left({min}_{xd}{,max}_{yd}\right)$, where ${min}_{xd}$ is minimum foot contour value along the x-axis, and ${max}_{yd}$ represents maximum foot contour value along the y-axis.

The coordinates of point t4 were determined in the following way: $t4\left({min}_{xd},0\right)$, where ${min}_{xd}$ represents minimum foot contour value along the x-axis, and the second coordinate is the zero value along the y-axis.

The next step was to determine the value of the angle *θ*, which was necessary to perform a geometric transformation of the rotation. In order to determine the value of the angle, it was necessary to know the slope of the lines p1 and p2, which can be seen from the equation:

(2) $$\tan\theta =\left|\frac{m_{2}-m_{1}}{1+m_{2}*m_{1}}\right|$$

Here, *m*_1_ represents the slope of the line p1, and *m*_2_ the slope of the line p2. The slope of the line p1 is calculated according to the formula:

(3) $$m_{1}=\frac{{max}_{yd}{-min}_{yd}}{{min}_{xd}-{x1}_{avg}}$$

The slope of the line p2 is calculated according to the formula:

(4) $$m_{2}=\frac{{max}_{yd}-0}{{min}_{xd}-{min}_{xd}}$$

Based on tan θ, it was simple to calculate the required angle. The obtained angle is in radians, so it was necessary to convert the angle value into degrees, based on the following formula:

(5) $$\theta \left[{}^{\circ}\right]=\frac{\theta \left[rad\right]}{\pi }*180{}^{\circ}$$

**Figure 5:**
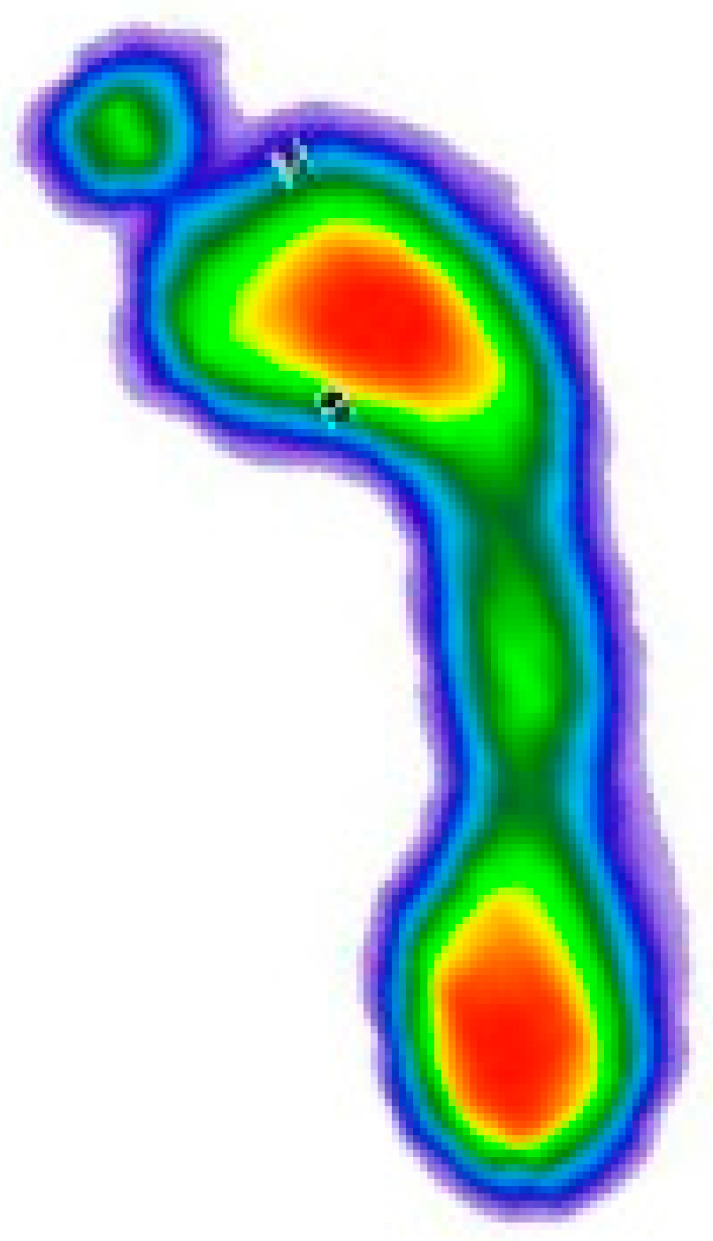
Foot rotated by angle *θ*. The color map of the pressures: red to dark green—from the area of the highest to the lowest level of pressure; blue—foot perimeter.

After determining the value of the angle, a geometric transformation of the rotation for the given angle was applied, which can be seen in Figure 5.

**Figure 6:**
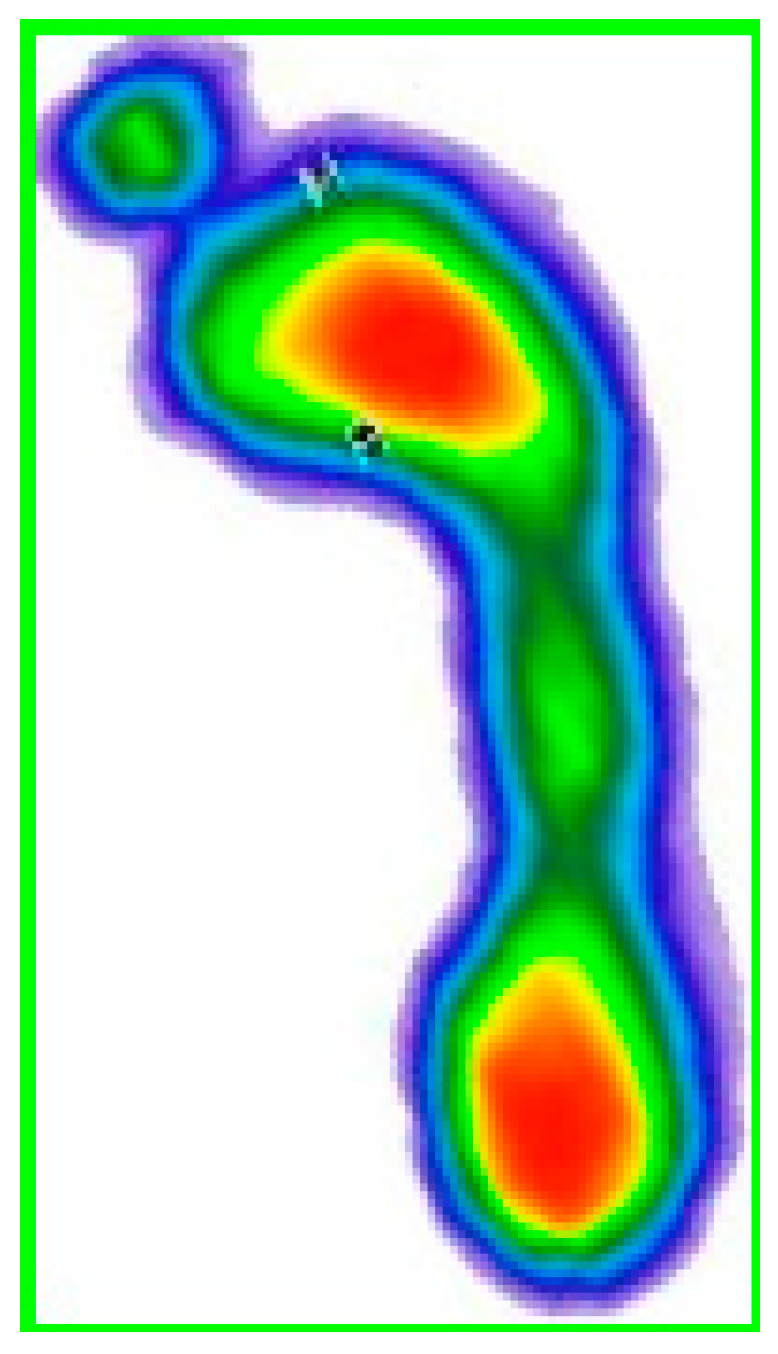
Contour detection and rectangle generation. The color map of the pressures: red to dark green—from the area of the highest to the lowest level of pressure; blue—foot perimeter.

Given that each person positions their foot in a specific way and has a different foot progression angle (*θ*), it was necessary to perform the previously described rotation of the foot for that angle, with the aim of obtaining maximally uniform data [20]. The contour detection and rectangle generation technique was again applied, which is shown in Figure 6.

The next step was applying techniques in order to remove toeprints, so that the arch index value could then be determined. Toeprints needed to be removed because they are not used to calculate the foot arch index, they transfer an insignificant percentage of one’s body weight to the ground, and they are extremely variable. A modification of the method introduced by Oliveira et al. was applied here [21]. 

The removal of toeprints was performed for a precisely defined value of the radius of the circle whose center is also the center of the contour of the entire foot. The center was generated based on the centroid, which represents the arithmetic mean of all points. In the context of image processing and computer vision, each shape is made up of pixels, and the center is a weighted average of all the pixels that make up that shape. The centroid was found via the moments of the image [22]. The radius (*R*) represents the length between the center of the contour of the entire foot and the point on the contour of the area with greatest pressure of the forefoot. The coordinates of that point correspond to the minimum value of the contour along the y-axis and the zero value of the coordinate system along the x-axis. The value of the radius is determined based on the Euclidean distance between the two previously mentioned points, according to the formula:

(6) $$R=\sqrt{{\left(0-C_{x}\right)}^{2}{-({min}_{yg}-C_{y})}^{2}}$$

Here, *C_x_* and *C_y_* are the coordinates of the center of the circle and, therefore, the center of the footprint of the entire foot, while ${min}_{yg}$ represents y-coordinate of the minimum value of the contour of the area of greatest pressure of the front part of the foot.

**Figure 7:**
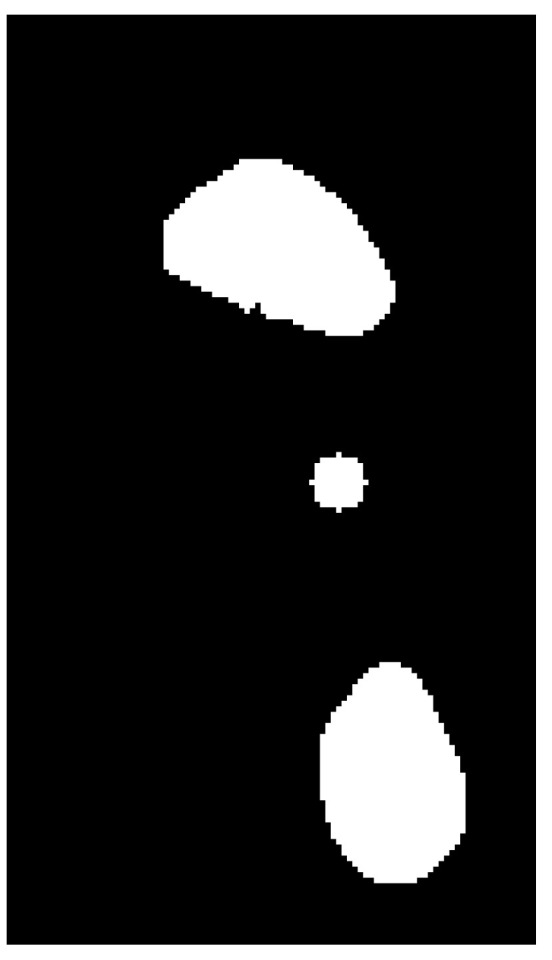
The regions of greatest pressure and the center of the contour of the entire foot.

The first technique is the segmentation (separation) of the desired regions, which are the areas of greatest pressure in the front and back of the foot. The representation of the selected regions and the center of the contour of the entire foot is shown in Figure 7.

**Figure 8:**
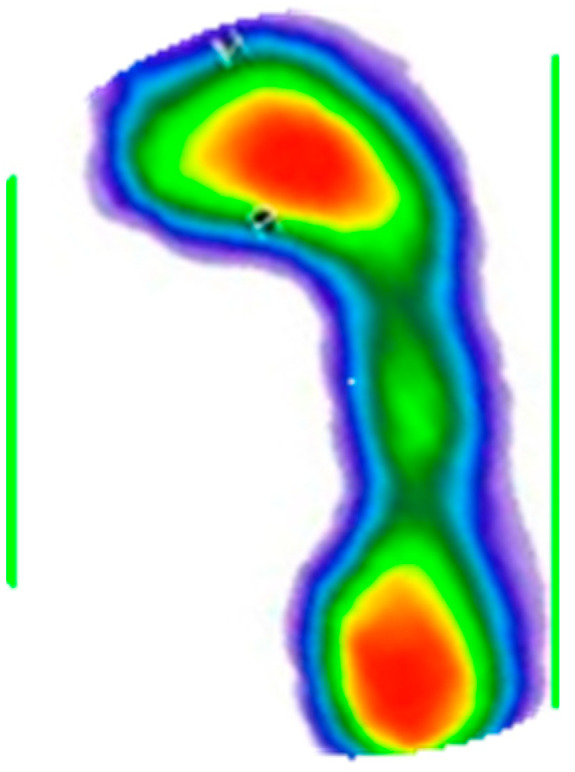
A footprint with the toeprints removed. The color map of the pressures: red to dark green—from the area of the highest to the lowest level of pressure; blue—foot perimeter.

Regions with the highest intensity of pressure are highlighted, which correspond to the red spectrum (warmer colors, such as red, are fields of high pressure on the ground). The segmented region (footprint with toeprints removed) can be seen in Figure 8.

**Figure 9:**
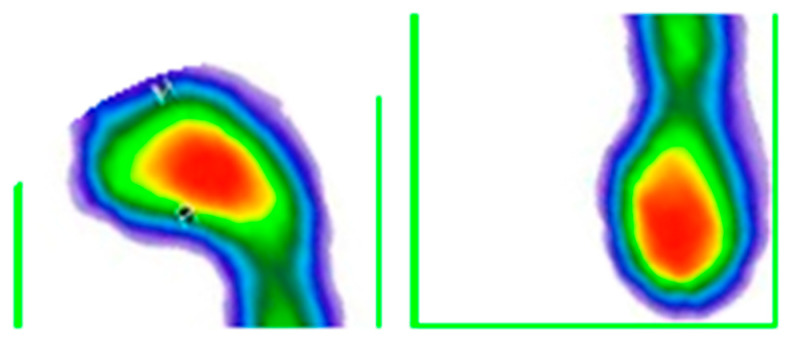
Upper (**left**) and lower (**right**) halves of the footprint. The color map of the pressures: red to dark green—from the area of the highest to the lowest level of pressure; blue—foot perimeter.

After the segmentation, an unwanted artifact in the form of the removal of a part of the heel print was observed; thus, the segmentation of the upper half of image 7 and the lower half of image 5 was applied (Figure 9).

**Figure 10:**
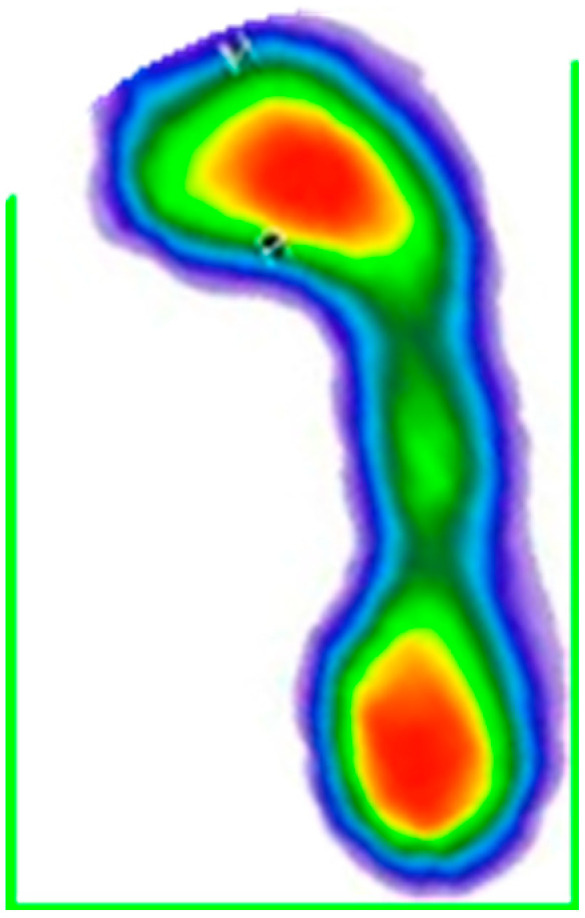
An image of a footprint without fingerprints and without heel artefacts. The color map of the pressures: red to dark green—from the area of the highest to the lowest level of pressure; blue—foot perimeter.

The concatenation (linking, merging) function was then applied, which resulted in an image without the aforementioned artifact (Figure 10).

**Figure 11:**
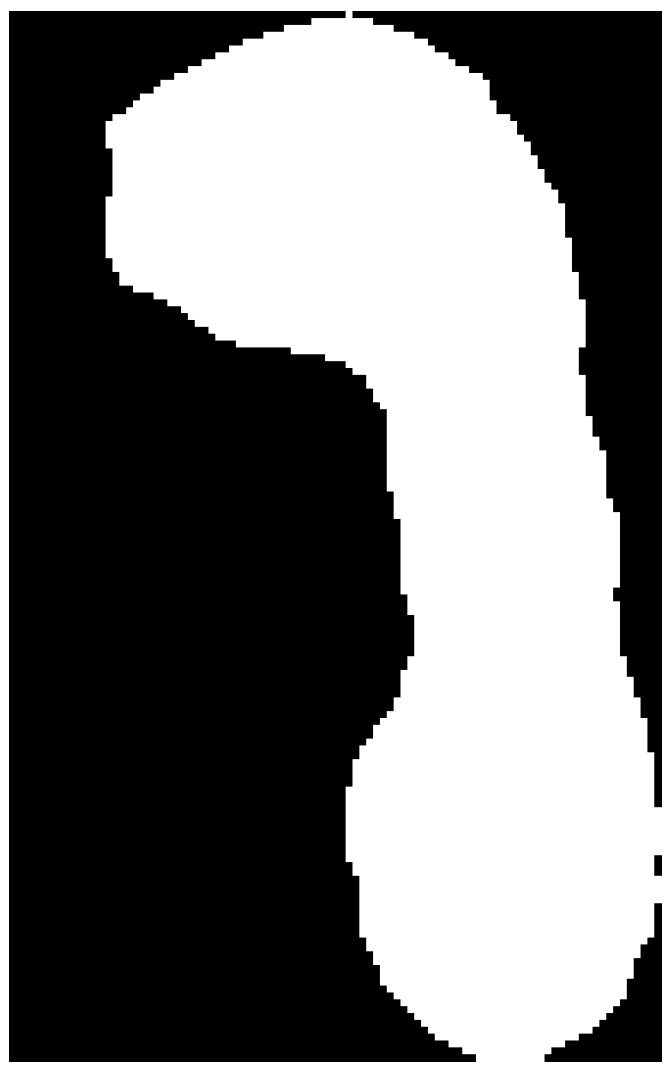
Image after the application of morphological operations of erosion and dilatation.

After removing the remaining traces of the contour and applying erosion and dilation operations, a black and white image was obtained (Figure 11). Erosion and dilation are morphological image-processing operations. Morphological image processing implies modification of geometric structures in the image. These operations are primarily defined for binary images but can also be used on grayscale images. Erosion removes the outermost layer of pixels in the structure, while dilation adds a layer of pixels to the structure [23].

**Figure 12:**
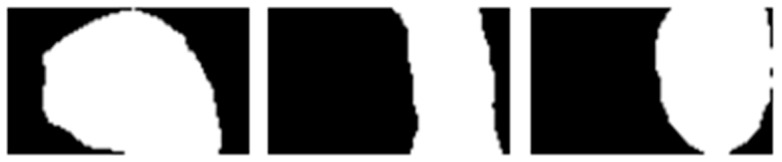
Display of the thirds of the image that are equal in height to one another; front (**left**), middle (**middle**) and rear (**right**) parts of the foot.

The last step was to divide the height of the image into thirds with equal heights, and the area of each third of the footprint (Figure 12, white region) was calculated.

The surface values are used to calculate the foot arch index (*AI*) according to the formula:

(7) $$AI=\frac{B}{A+B+C}$$

The surface of the middle part of the foot is marked with *B*, the surface of the front part with *A* and the surface of the back of the foot with *C*. Therefore, *AI* represents the ratio of the area of the middle part of the foot to the area of the whole foot. It is a dimensionless quantity.

Based on the foot arch index (*AI*) value, foot deformities can be classified. If *AI* < 0.17, it is a hollow-foot-type deformity (*pes cavus*), and if *AI* > 0.28, it is a flat-feet-type deformity (*pes planus*). When 0.17 ≤ *AI* ≤ 0.28, it is a healthy foot without either of these two deformities [15,20].

## 3. Discussion

For the foot on which the entire previously described labeling method was applied, an arch index value of 0.27 was obtained, which represents a healthy foot, and indicates the accuracy of the method. Our paper is an attempt to objectify diagnoses of foot deformities; we listed and applied the techniques of segmentation, geometric transformation, contour detection and morphological image processing in order to calculate the foot arch index, based on which information on the type of foot deformity was obtained. 

The method that was developed and applied in this study gave a result that was consistent with the clinical findings, which were that the patient had a healthy foot (with the absence of flat or hollow feet); therefore, this indicates the accuracy of the method itself [15,16,17,18]. However, this model requires further improvement and optimization, because the results of the segmentation techniques may vary when the images are not consistent. Moreover, manually cutting the images from the .pdf document was the only process that was not automated; thus, it is the main limitation of the technique. 

Additionally, regarding foot deformity classification, a similar labeling method was applied in the research of the author Chae J. et al., in which their results were then used to create machine learning algorithms [20]. Another recent example of a machine learning approach in orthopedics was the prediction of the risk of diabetic foot ulcers from plantar pressure images, with a procedure similar to ours [24]. Furthermore, the estimation of various walking intensities based on wearable plantar pressure sensors was performed using an artificial neural network, another machine learning method. Namely, that contemporary study was in line with our technique since it used plantar pressure images and the data-labeling method [25]. Finally, artificial intelligence is useful in other fields of medicine as well, such as the prevention of intraoperative anaphylaxis [26]. Overall, the main point of the methods presented here and elsewhere is the necessity of using artificial intelligence to remove the manual steps in image processing and minimize human error.

## 4. Conclusions

The labeling method presented in this paper provides a basis for the further optimization and development of a system for classifying foot deformities based on machine learning techniques. Furthermore, this technique could provide better individualization of therapeutical procedures in orthopedics, though the reliability of the method needs to be verified in future studies. It is our expectation that gaining a deeper understanding of potential applications will assist orthopedy practices in preparing for the future and achieving increased efficiency and performance improvements.

## Data Availability

The data presented in this study are available on request from the corresponding author.
